# Automatic detection of image manipulations in the biomedical literature

**DOI:** 10.1038/s41419-018-0430-3

**Published:** 2018-03-14

**Authors:** Enrico M. Bucci

**Affiliations:** 1 Temple University, Philadelphia, PA USA; 2 Sbarro Health Research Organization, Philadelphia, PA USA

## Abstract

Images in scientific papers are used to support the experimental description and the discussion of the findings since several centuries. In the field of biomedical sciences, in particular, the use of images to depict laboratory results is widely diffused, at such a level that one would not err in saying that there is barely any experimental paper devoid of images to document the attained results. With the advent of software for digital image manipulation, however, even photographic reproductions of experimental results may be easily altered by researchers, leading to an increasingly high rate of scientific papers containing unreliable images. In this paper I introduce a software pipeline to detect some of the most diffuse misbehaviours, running two independent tests on a random set of papers and on the full publishing record of a single journal. The results obtained by these two tests support the feasibility of the software approach and imply an alarming level of image manipulation in the published record.

## Introduction

In a set of drawings dating 13th March 1610 published on the “Sidereus Nuncius”, Galileo represented the uneven curve of the sun’s light over the moon disc, as seen only in January of the same year using his telescope^1^. The intent was to prove that the moon surface was rough, with several differences in elevation, in contrast to the idea prevalent at the time of a smooth, perfect sphere. This is a good example of the conscious usage of a series of images to document a scientific observation and to prove a scientific hypothesis, a common practice in several domains of science. Given the complexity of the subjects to be represented, however, in the field of life sciences only few scientists with excellent drawing skills (or having access to gifted artists) could successfully and universally propagate their findings using images— think for example of Haeckel’s embryos or of Darwin’s orchids. It was only after “objective” photographic reproduction of experimental outcomes was routinely available, that using images to represent the outcome of a biological experiment became a method accessible to anyone; a method perceived to be as objective as any other experimental set-up, so that in many cases images produced by dedicated apparatuses became the results to be analyzed, qualitatively and quantitatively, to prove a given hypothesis. This fact led to a proliferation of images published in the biomedical literature, where photographs are used to document experimental results, as opposed to abstract graphs and graphical arts used mostly to summarize mathematical quantities or to represent an experimental set-up or a theoretical model. The status of relative “objectivity” attributed to photographic documents was however severely challenged in the transition from classical photography to digital imaging, because the same software used for producing and analyzing digital images was very early used to retouch the images to be published. While this can be acceptable in principle—for example, intensity calibration of a digital image can be required for a quantitative analysis—it is also true that image manipulation aiming to deceive the readers of a scientific paper became extremely easy. The once difficult photographic retouching is today technically available to anyone; thus, an easy prediction would be that illicit manipulation of scientific images should be highly prevalent. In particular, once the original obstacle (i.e., technical feasibility) has been lifted, there are certain conditions that would lead to a higher number of misconduct cases connected to image manipulation, namely:

1. the manipulation confers some strong advantages to the person committing it;
2. the probability of being discovered is low;
3. even after an actual fraud is discovered, the consequences for the offender are mild if any.

Indirect evidence for the hypothesis that fraudulent image manipulations are indeed increasingly common comes from the US Office for Research Integrity (ORI) database. In fact, since the introduction of Photoshop in 1988, the number of ORI cases with questioned images has been growing exponentially.^2^ However, image manipulations that surfaced in ORI cases are by definition originating from a tiny selection of research groups—only cases involving US Federal Funding are reported to and considered by ORI—and, even for the population considered, ORI cases are suspected to be only the tip of the iceberg.^3^ In recognition of this problem, we thus decided to measure the actual extent of suspect image manipulation in the biomedical literature by performing an unbiased, automated analysis of a large image sample obtained from recent scientific publications, supplemented by expert analysis for verification of the findings. To this aim, we tweaked some home-made software with available open-source and commercial tools, to get an efficient pipeline for the extraction and processing of images from the scientific literature on a bulk scale.

## Type of image manipulations considered and instruments selected for the analysis

One of the most debated questions in the field of scientific misconduct involving images is the necessarily arbitrary definition of what is acceptable and what is not. Beside the general idea that manipulations that aim at deceiving the reader, concealing data features, or fabricating whole or parts of an image are all examples of misconduct, there are few if any clear-cut guidelines. We started from the ORI guidelines as reported in the ORI website at the time of writing this manuscript.^4^

In particular, we considered the following evidence of potential misconduct:

1. Cloning objects into an image, to add features that were not present in the first place, taking the cloned object from the same or a different image;
2. Reusing an image or a “slightly modified” version of the same image in the same paper without an explicit mention of it. Two or more images are considered as a “slightly modified” version of a single image if their difference is restricted to a small, discrete region (not larger than 5% of the total area expressed in pixels), or if they differ only in scale, rotation, linear stretching, cropping, contrast, or brightness (in any combination);
3. Reusing an image or part of it from a previous paper, including reusing a “slightly modified” version of a previously published image (in the same sense as for point 2).

This list is intentionally restricted to a fraction of what is technically possible to detect, because proving any of the above-mentioned image manipulation strongly implies a scientific misconduct case.

Specifically, point 1 corresponds to data fabrication—no original experiment for the published image exists.

Points 2 and 3 include cases that range from image plagiarism, if the involved images are presented in the same way (e.g., they are labelled in the same way and they are referred to the same experiments, discussed in the same way), to falsification, if they are presented as referring to completely different experiments (e.g., they are labelled differently and refer to physical objects which are not the same).

## Investigation of a random set of Open Access papers

In a first experiment, we considered the open source papers released by PubMed Central^5^ (PMC) in January 2014. Assuming a global population of 30,000,000 of papers, to ensure that the results were representative (error level ±5% with 99% confidence), we included in this sample a number of papers equal to more than twice the minimum requested sample size (which would be 664). In this way, we could balance for the presence of up to 50% of irrelevant papers (review, letters, image-free papers etc.). The sample thus included 1364 papers randomly selected from PMC, from 451 journals. After automated extraction and filtering, this set gave 4778 images annotated by the software pipeline. The processing time on a Xenon E5 exacore equipped with a 30 Gb RAM was about 30 min.

Out of the 1364 examined papers, we discovered 78 papers (5.7% of the total) from 46 different journals (10.2% of the total, average IF = 4.00, ranging from 0.11 to 9.13) containing at least one instance of suspected image manipulation. To see whether any of the retrieved papers was known to contain any problem, we checked twice on the anonymous post-publication peer review site PubPeer (www.pubpeer.com): once at the time of the first analysis (March 2014) and once at the time of preparation of this manuscript. None of the identified papers was found among those discussed by PubPeer. It is to be seen whether the site community will detect problems in the identified papers in the next future.

As for the type of manipulated images, the vast majority of the identified papers contain manipulations of gel electrophoresis images (*n* = 65, i.e., 83% of flagged papers contain at least one manipulated gel image). Given the fact that part of our pipeline was specifically designed to identify cloning of bands and lanes in gel images, this result is hardly surprising. However, if we refer this number to papers containing at least one image of a gel electrophoresis experiment (*n* = 299), we obtain that 21.7% of this subset do contain a potential ORI policy violating manipulation involving gel images—which appears to be a high incidence per se. This particular finding comes as an experimental verification of suspicions raised on the extensive manipulation of gel-electrophoresis images by Marcus and Oransky^6^ among others, and it is consistent with the easiness with which such manipulations can be produced and can escape human visual inspection.

The affected journals that yielded more than one paper for the analysis are reported in the Table 1, sorted by number of papers included in the sample (examined papers). The absolute number of potentially manipulated papers and the corresponding ratio over the total is reported for each journal.

**Table 1:** Analysis of a random sample of PMC journals. For each journal, the number of examined papers, the number of potentially manipulated papers and the corresponding ratio of manipulated papers is reported. Journals with only 1 examined paper are not reported in the table

Examined papers	Suspicious papers	Ratio	Journal
127	18	0.14	PLoS One
59	5	0.08	Nucleic Acids Res
55	3	0.05	Exp Ther Med
55	1	0.02	Oncol Lett
32	2	0.06	Sci Rep
32	1	0.03	Yonsei Med J
24	1	0.04	J Radiat Res (Tokyo)
22	2	0.09	J Exp Bot
18	3	0.17	J Biol Chem
15	1	0.07	PLoS Genet
14	3	0.21	Oncol Rep
14	1	0.07	Int J Mol Med
12	1	0.08	Dis Model Mech
9	1	0.11	Mol Cell Proteomics
8	1	0.13	Br J Cancer
7	2	0.29	PLoS Pathog
5	1	0.20	Clin Ophthalmol
4	1	0.25	Mol Cell Biochem
3	2	0.67	Biochem J
3	1	0.33	J Chromatogr
3	1	0.33	Mol Cancer
2	2	1.00	Theranostics
2	1	0.50	Indian J Lepr
2	1	0.50	BMC Complement Altern Med
2	1	0.50	J Exp Clin Cancer Res
2	1	0.50	Mol Vis
2	1	0.50	J Clin Biochem Nutr
2	1	0.50	Mol Biol Rep
2	1	0.50	BMC Neurosci
2	1	0.50	J Biol Inorg Chem
2	1	0.50	BMC Cancer
2	1	0.50	Plant Mol Biol
2	1	0.50	PLoS Negl Trop Dis

Of note, we checked whether there is any correlation between the ratio of manipulated papers and the IF of the affected journal (2012 values), but we could find no evidence for it. In this respect, at least in the examined sample, we could neither find that higher IF guarantee more stringent checking procedures, nor that journals having higher IF are target of more manipulations.

We then tested whether the amount of image manipulations found in each journal correlates with the number of retractions already published by that journal. This possibility follows from assuming that image manipulation is highly prevalent among scientific misconduct cases—which is indeed true for claims examined by ORI^2^—and that (when discovered) it results in a retraction, so that journals were image manipulations are highly prevalent should also retract more papers than others. To test whether this correlation exists, at least in the limited sample examined in this paper, we isolated from our set those journals which:

1. were represented by at least 10 papers included in our initial set;
2. were found to have at least 1 manipulated paper included in our set;
3. had at least 1 paper dubbed as retracted by PubMed.

**Fig. 1:**
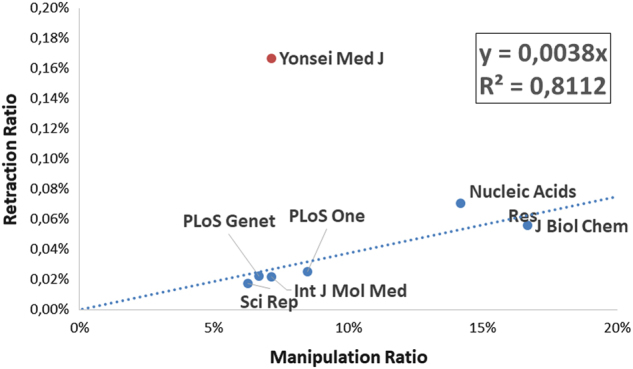
**Linear correlation between the retraction rate and the rate of manipulated images found in published manuscripts, as obtained in the examined random sample of journals considered in this paper**

Seven journals satisfied all the above-mentioned conditions. We thus compared the ratio of manipulated images found in the sample to the ratio of retracted papers (number of retracted over total published papers). The result is exemplified in Fig. 1.

A strong linear correlation is observed between the Image Manipulation Ratio and the Retraction Ratio for all journals but the Yonsei Medical Journal, which appears to have by far more retractions than expected. An examination of the 6 retractions retrieved for this journal (at the time of preparation of this paper) may clarify why: 4 retractions were due to text plagiarism, 1 to intellectual property issues, and 1 is due to undisclosed reasons. It appears that, for this journal, retractions do not correlate with image manipulations; whether this fact is due to lack of detection or to dismissal of the corresponding manipulation claims by the editorial board remains to be ascertained. However, it holds true that, for 6 out of the 7 journals examined, the image manipulation rate appears to correlate with the retraction rate. As shown in the previous graph, for the journals considered, retractions totaled a mere 0.38% of papers containing potentially manipulated images. However, for the same journals, an examination of retractions or corrections reveals that, for those cases where enough information is disclosed, a substantial amount of retractions is indeed due to image manipulations of the kind discussed in this paper. Table 2 shows the number of retractions which were at least in part caused by image manipulations (as of May 2015).

**Table 2:** Analysis of paper retracted for the journals reported in figure 1. See figure 1 for further details

Journal	Retracted papers	Retractions due to image manipulations	Retractions due to other problems	Unknown reason
Nucleic Acid Res	8	2	6	0
J Biol Chem	26	15	0	11
PLOS One	37	10	27	0
PLOS Genet	2	1	1	0
Int J Mol Med	2	0	1	1
Sci Rep	4	0	2	2
Yonsei Med J	6	0	5	1

On average, image problems are reported in about 40% of the retraction notes detailing the reasons for paper withdrawal. Therefore, it appears that the discrepancy between retraction rates and manipulation rates is mainly due to a detection problem, not to the dismissal of claims by the editorial boards.

**Fig. 2:**
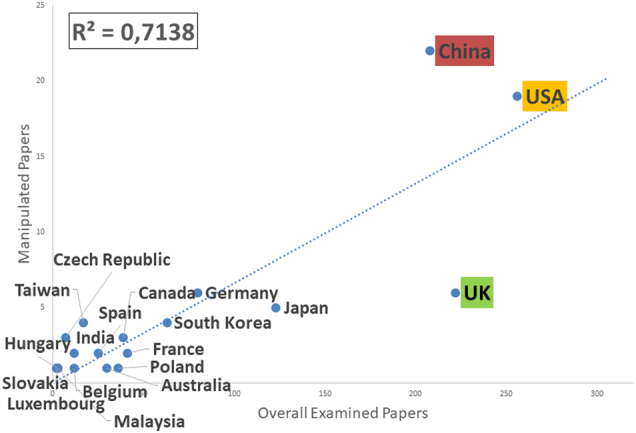
Number of papers containing manipulated images as a function of the overall number of papers examined for each nation included in the analyzed random sample. A paper is attributed to a given nation according to the nationality of affiliation of the institution of the corresponding author

We next examined the distribution of manipulated papers by country. We assigned each paper to a country according to the location of the corresponding author’s institutions. The original sample contained papers from 69 countries, with an average of 21 papers per country (standard deviation = 49, range = 1–256). Figure 2 reports the number of problematic papers as a function of the total number of examined papers for each country.

Considering the first three countries sorted by number of examined papers, groups from China (and to a lower extent USA) produced more manipulated papers than expected, while UK groups produced less.

**Fig. 3:**
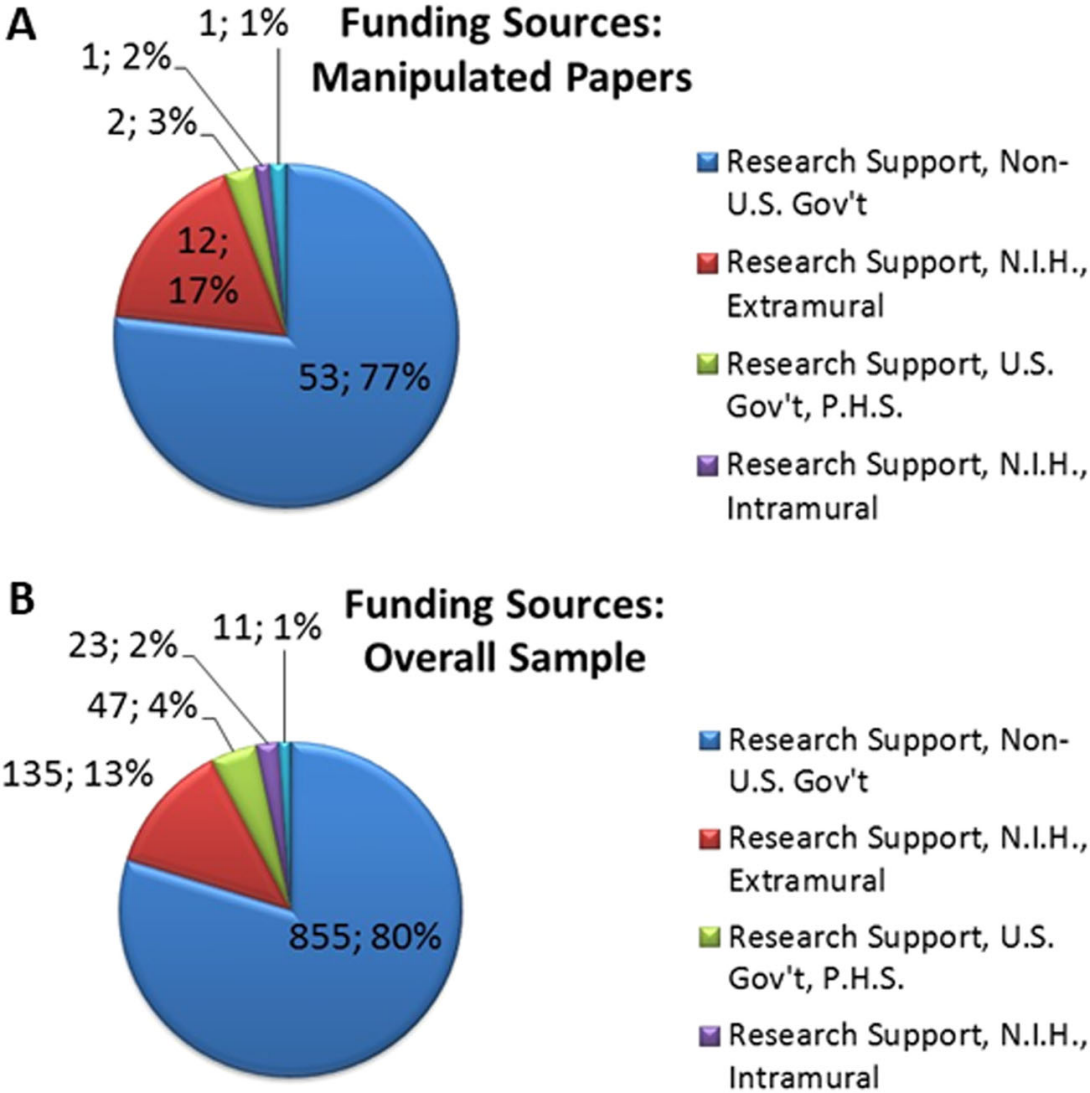
**Disclosed source of funding for the paper containing manipulated images (upper pie) and for the overall examined random sample (bottom pie)**

Eventually, in an attempt to evaluate the potential economic impact of the 78 papers containing some problematic images, we retrieved the funding information provided by PubMed for each of the included papers. While this information is only partial—there is a variety of obligations in disclosing funding sources, which depends on National legislations—we could nonetheless assess the minimal economic impact by examining the disclosed information. The number of problematic papers per funding source according to PubMed is reported in the upper pie graph in Fig. 3. The bottom pie graph represents the distribution of funding sources for the overall test sample (information available for 926 papers out of 1364).

First, by comparing the funding source distribution in the two pie charts, we may notice that there is no specific enrichment in the set of manipulated papers. This means that there is not a “preferred” funding source for manipulated papers.

As for the 78 papers containing manipulated images, the total number of papers, which disclosed some funding information, is 53, with some of the papers having more than one funding source (69 grants reported as a funding source). While the identified paper manipulations are not necessarily connected to misconduct or fraud in a scientific project, the corresponding grant values allow the estimate of a lower bound for the money potentially lost in bad science. For example, if we consider that the average value of extramural NIH research projects is above $400,000^7^, then the overall value for the 12 NIH projects which produced 12 problematic papers (red portion of the pie in the preceding graph) is greater than $4,800,000, in agreement with an independent estimate that was recently published.^8^ It should be noted that, while detecting manipulations in papers cannot prevent the loss of money invested in the corresponding projects (since it already happened), it can however prevent these papers to be used in further grant requests, and, if used to screen at that level, can be used to assess the quality of all data—including unpublished one.

## Uncovering time trends: investigation of the journal *Cell Death and Disease*

To further test our pipeline, we would approach the analysis of a single journal, examining all its published papers, instead of a sample of different journals like in the previous example.

This allows to follow the temporal spreading of the manipulations, to see whether there is a growing trend or a nearly constant rate of manipulation. Moreover, if the yearly acceptance rate for a journal is known, it is possible to see whether a direct correlation exists between published manipulated data and easiness for a paper to get published. As a last point, by looking at the entire dataset of images published by a journal, image reusing may be easily spotted, adding a further layer to detectable misconduct.

We selected as a representative target the journal *Cell Death and Disease* (CDDIS), published by the Nature Publishing Group (NPG), and focused on the 1546 papers published in the period 2010–2014. Overall, we found 8.6% of papers to contain manipulated images, which is well in the range found for the PubMed Central sample.

**Fig. 4:**
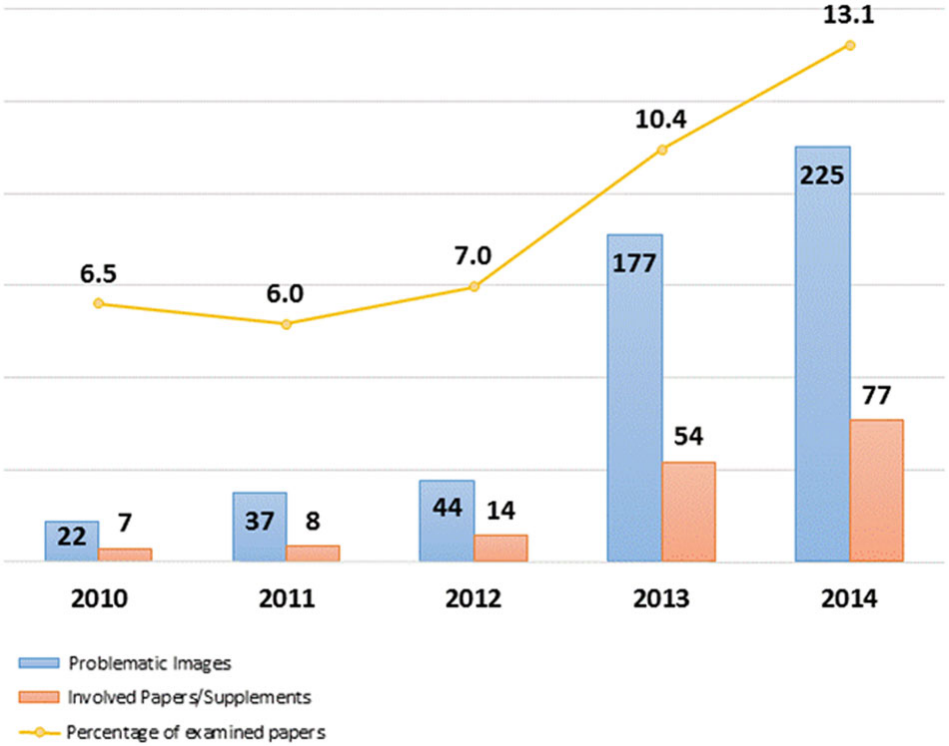
Yearly rate of published papers containing at list one manipulated image (orange line) for the journal *Cell Death and Disease*. The yearly number of retrieved manipulated images and the number of papers containing them is also reported (blue and red bars, respectively)

However, if one looks to the temporal evolution of the yearly percentage of manipulated papers, a growing trend is immediately evident, with a percentage of manipulations exceeding the PMC range in the last 2 years examined (Fig. 4).

While for CDDIS we observed a growing trend in image manipulation, from published data it appears that during the same period the acceptance rate slightly decreased (from above 50% to about 40%).^9^

However, the overall number of papers submitted increased substantially, from about 100 papers in 2010 to more than 1000 in 2014. The I.F. of the journal during the same period has been stable or it slightly increased; however, the submission quality was apparently affected by an increase in potentially manipulated images, which again confirms that these two parameters are not correlated.

While from a publisher perspective I.F. and submission growth are the hallmark of a successful journal, our data point to the fact that, without some extra checking of the manuscript quality, it is not possible to ensure that a highly successful journal (in terms of its reception by the public) is free of a substantial number of manipulated images.

Another interesting result, which is not caught by the preceding figures and tables, is that of image reusing in different papers published by the same journal. By looking to papers published by CDDIS in 2014, we discovered that 20 contained images previously published by the same group of authors. While self-plagiarism could have easily been spotted by an automated procedure relying on a database including all images published by a journal, referees of a paper submitted in 2014 might have never seen the reused images before or might have dedicated less time to the revision process, due to the aforementioned increase in the number of submitted manuscripts. To draw any conclusions, one should compare my analysis with the analysis of a journal of similar level, that in the same period has not increased the number of articles.

## Conclusions

We run a scientific literature analysis to check for image manipulations. While our approach suffers from a few limitations in scope, being restricted to the detection of only a tiny amount of possible data manipulations, we discovered that about 6% of published papers contain manipulated images, and that about 22% of papers reporting gel electrophoresis experiments are published with unacceptable images. This last figure might be compared to the result of recently published independent study^10^, which examined a set of randomly selected papers in basic oncology and found 25% of them containing manipulated gel images.

On a larger dataset, using flagging criteria that appear to be quite like those assumed in this paper, Bik and coworkers found 3.8% of manually examined papers to include at least one figure containing an inappropriate image manipulation. Looking at single journals in the same sample, this percentage ranged from 0.3% in *Journal of Cell Biology* to 12.4% in *International Journal of Oncology*, albeit the examined samples for different journals were very different in size^11^. Again, the manipulation rate found independently on a different sample of papers appear compatible to what has been automatically detected using our procedure in a different sample.

Moreover, our study allowed for the first time, albeit in a limited sample, to establish a correlation between the number of manipulated images in a journal and the number of retractions issued by that journal: with some notable exception, the measured ratio of manipulations is a proxy for the retraction rate. This is also confirmed by the fact that the retraction notes refer in a substantial number of cases to image manipulations; however, the number of published papers containing manipulated images exceeds by orders of magnitude the number of retracted papers, pointing to a detection problem on the journal side.

As for the details of our analysis, in contrast with previous proposed hypotheses and some published results^11^, we could not find any correlation between a journal impact factor and the ratio of manipulations detected in our sample, but we do identified China as a country producing more problematic papers than average (in agreement with statistics on different kinds of misconduct, such as plagiarism) and UK as a country producing less. This last result might be related to the limited period considered by our analysis (a single month in 2014), and needs to be confirmed before trusted; both the higher prevalence measured for China and the lower measured for UK do however agree with recently published data^11^.

Eventually, by focusing on a single journal from the Nature Publishing Group, we could unearth a temporal growing trend for potential misconduct connected to image manipulation, which sounds as an alarm bell for any journal.

In conclusion, we want to stress here the fact that, being such a prevalent form of misconduct, image manipulation should and could be faced by journals, and no delay can be allowed anymore, nor can it be justified by pretending the analysis is too complex or long to run.

At the same time, academic and scientific institutions should implement procedures to properly handle allegations of image manipulations, using software tools as a source of unbiased flagging and screening, before human assessment leads to conclusion about any potential misconduct; this process indeed is initiated in some large scale research institution^2^.

## Materials and methods

We designed an automated pipeline able to extract all images from a set of papers and perform several tests on the images aiming to assess any evidence of the type described in the previous section. This pipeline includes the use of the following open source, commercial and in-house pieces of software:

1. A pdf converter, to extract single pages from papers and save them as jpg files (we used a home-made software, but there are several open source tools that can be used for the same scope).
2. An in-house developed software, dubbed ImageCutter, to perform the extraction of image panels from each page.
3. A specific gel-checking routine, named ImageCheck, to uncover cloning of image portions elsewhere in the same or in different image panels.
4. An image duplication tracking software, to check for image panel duplication in the same or in different papers.

The first step in the procedure starts from a set of pdf files corresponding to a collection of papers to be analyzed (pdf conversion step). From each pdf file, an ordered set of jpg files is generated, each jpg file corresponding to an entire page of the original pdf file.

**Fig. 5:**
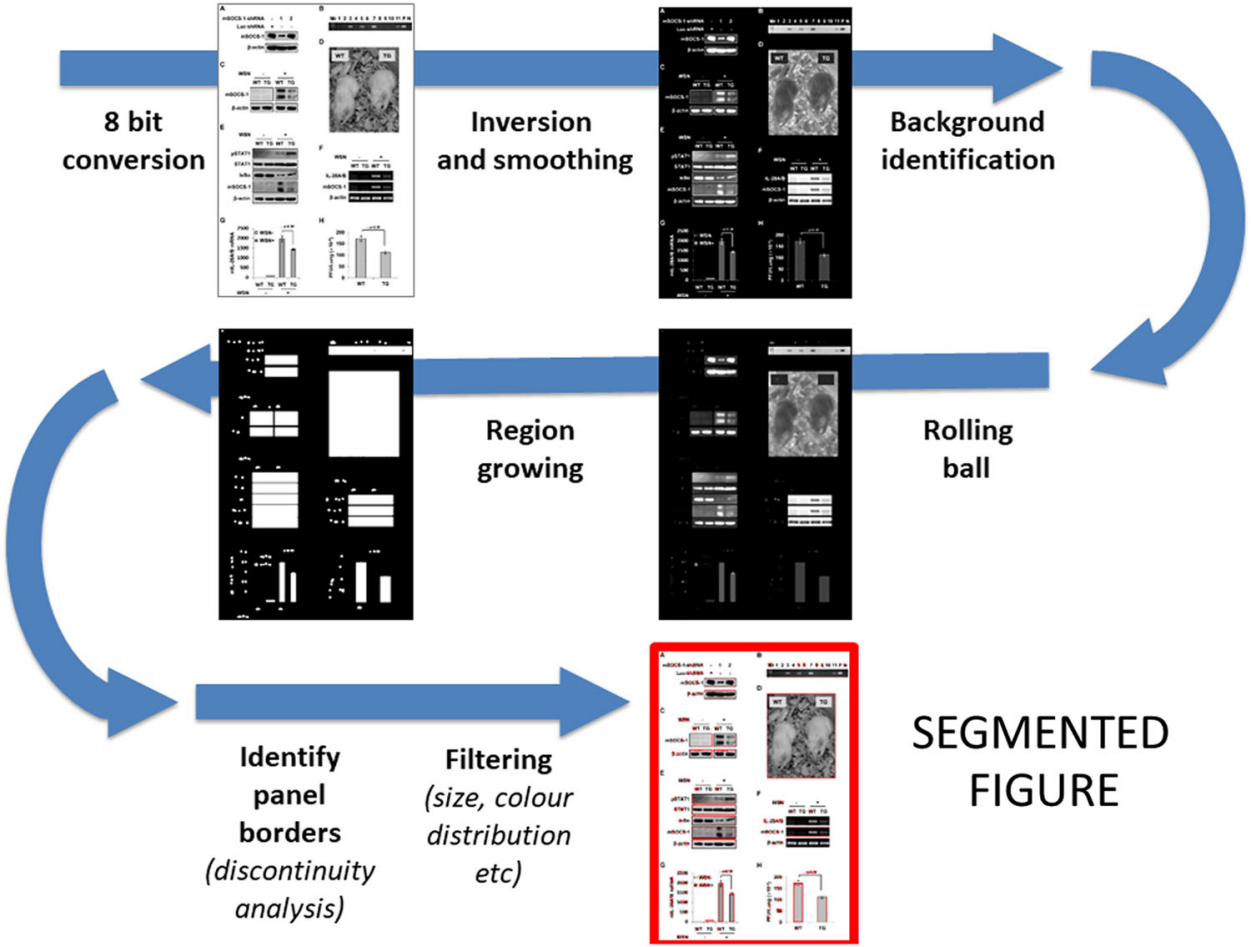
Workflow to extract image panels from a pdf file, corresponding to the automatic procedure adopted by the Java software ImageCutter. JPEG versions of each page in the pdf file are changed to 8-bit, gray level images. Assuming a white background, page images are then inverted, smoothed and used for a segmentation step adopting the rolling ball procedure. To avoid over-segmenting, a region growing step increasing each segmented region is performed; if two segmented regions are joined after moderate area growing, they are considered as a single object to segment. After that, borders of the segmented object are refined by identifying abrupt changes in gray level intensity, assuming all objects to segment are rectangular. After segmentation, those objects with predefined properties (e.g., uniform color and small size) are discarded, while all the others are passed as image panels to the following routines

The second step consists in the extraction of image panels from each page of any paper included in the original collection (panel segmentation step). Starting from the jpg files representing all the pages of the target papers, image panels are automatically identified and cropped using our in-house software ImageCutter. Ideally, an image panel is any portion of a page, which corresponds to a single graphical element—e.g. the photo of a single Petri dish or a single western blotting membrane. Small graphical arts—including, e.g., mathematical formulas, logos, or other small graphical objects, are filtered based on size of the generated image (images not larger than 10 Kb are eliminated). The automated workflow corresponding to this step is schematically represented in Fig. 5.

At this point, we have two sets of images:

1. The page set (jpg files corresponding each to a single page from a paper).
2. The image panel set (jpg files corresponding to single graphical elements incorporated into the figures of a paper).

The first set is used to check for cloning of specific image portions from the original location elsewhere in the image, as previously described—i.e., to detect a specific type of data fabrication. The second set is used for detecting image panel reusing—i.e., for finding out a type of plagiarism and a specific type falsification.

The third step consists in looking for cloned image portions. While changes in background, or intensity discontinuity or other subtle evidence can reveal the splicing of objects into images, to reach the absolute certainty of a cloning event one must find the source of the cloned object. This is an easy task when the object is present twice or more in the same or in different image panels—i.e., when one or more copies of the same graphical feature are detected in a figure, which is not expected to contain self-similar regions.

What kind of images are more often manipulated in this way? To address this question, we performed an overview of the biomedical publications retracted for image manipulation by looking at all open-source retracted publication in the PMC collection. In agreement with previous findings^6^, we realized that very often illicit cloning of portion images happens in figure depicting fictitious western blotting experiments (or other sorts of gel-electrophoresis experiments). An image documenting the result of a gel-electrophoresis experiment (including western blots) consists in a rectangular area, which should present a noisy background (either dark or light) and some prominent elliptical or rectangular spots (called bands, dark or light in opposition to the background), arranged in several columns (the gel lanes). The relative dimensions and the intensity of some bands in specific positions represent the expected outcome of an enormous variety of biomedical experiments, which is one of the reason why the technique is so popular among researchers (the other being its relative inexpensiveness). Fabrication of gel-electrophoretic images, on the other hand, is quite a simple process, and usually consists in the addition of some bands to a realistic background, to simulate the outcome of a given experiment. Given the large diffusion of the technique, as well as its prevalence in alleged cases of fabrication, we decided to tailor our routine toward the detection of fabricated gel-electrophoresis images. In particular, images corresponding to the jpg versions of each page from pdf files (the above-mentioned set 1) were used to feed a routine aimed to detect gel features cloned into a single gel or among different gels reported in a paper page. To achieve this, our software ImageCheck was set up to look for typical gel features in image panels, i.e., rectangular images, preferably in grey scale (or with a relatively low number of colored pixels), containing either a dark or a clear background and several internal spots. After a segmentation step, if their area did not differ by more than 10%, the software compared each possible couple of spots by reciprocal alignment (using their respective center-of-mass and allowing a minimal shifting in every direction). A couple of spots were flagged if after alignment (checking also for 180° rotation and/or a mirror transformation) a pixel-by-pixel intensity subtraction resulted in a difference lower than 2% of the normalized average area of the two spots (i.e., the sum of their area divided by 2). The 2% threshold was selected according to the R.O.C. analysis performed as described in the supplementary section.

Once all the gel images contained in the papers under investigation had been checked for cloned features, all image panels contained in set 2—representing a gel or any other type of scientific image—were used to investigate image reuse. To this aim, we originally used the commercial software “Visual Similarity Duplicate Image Finder Pro” (MindGems Inc), setting a threshold of 95% for similarity (the default value by the software producer) and allowing for intensity differences, vertical and horizontal stretching, mirroring, and 180° rotation. Of note, during the preparation of this paper, several free alternatives emerged to the commercial solution we used; however, we could not (yet) find anything as quick or effective as the originally selected tool, which was apparently developed for helping professional photographer to find duplicates in their large collections of images.

### Box 1: ORI and its investigations

The Office of Research Integrity (ORI) oversees and directs Public Health Service (PHS) research integrity activities in US on behalf of the Secretary of Health and Human Services except for the regulatory research integrity activities of the Food and Drug Administration.

It handles about 30 cases per year, based on allegations that (a) involve US-based laboratories and (b) involve work funded with public US money. Allegations led to finding of misconduct about in one-third of times (on average, 10 report of misconduct findings per year) and may lead to different administrative or even penal sanctions. (Source: Zoe Hammatt, presentation at the 4th World Conference on Research Integrity, Rio de Janeiro, 1 June 2015).

Around 70% of ORI cases involve image manipulations.^1^

## Electronic supplementary material

Supplementary Figure legend(DOCX 11 kb)

Supplementary Figure 1(TIF 324 kb)

Supplementary material(DOCX 19 kb)
