# Supplementary material for: Automatic detection of image manipulations in the biomedical literature
Source: Cell Death Dis. 2018 Mar 14;9(3):400. doi: 10.1038/s41419-018-0430-3 (PMC5852055; doi:10.1038/s41419-018-0430-3)
Supplement: Supplementary file 1 — Supplementary Figure legend(DOCX 11 kb) [file 41419_2018_430_MOESM1_ESM.docx]

**Figure S1.** R.O.C. Analysis to evaluate the precision of the proposed method on a sample of manually examined scientific images. Details in the supplementary section.
