# Supplementary figures and images for: Automatic detection of image manipulations in the biomedical literature
Source: Cell Death Dis. 2018 Mar 14;9(3):400. doi: 10.1038/s41419-018-0430-3 (PMC5852055; doi:10.1038/s41419-018-0430-3)

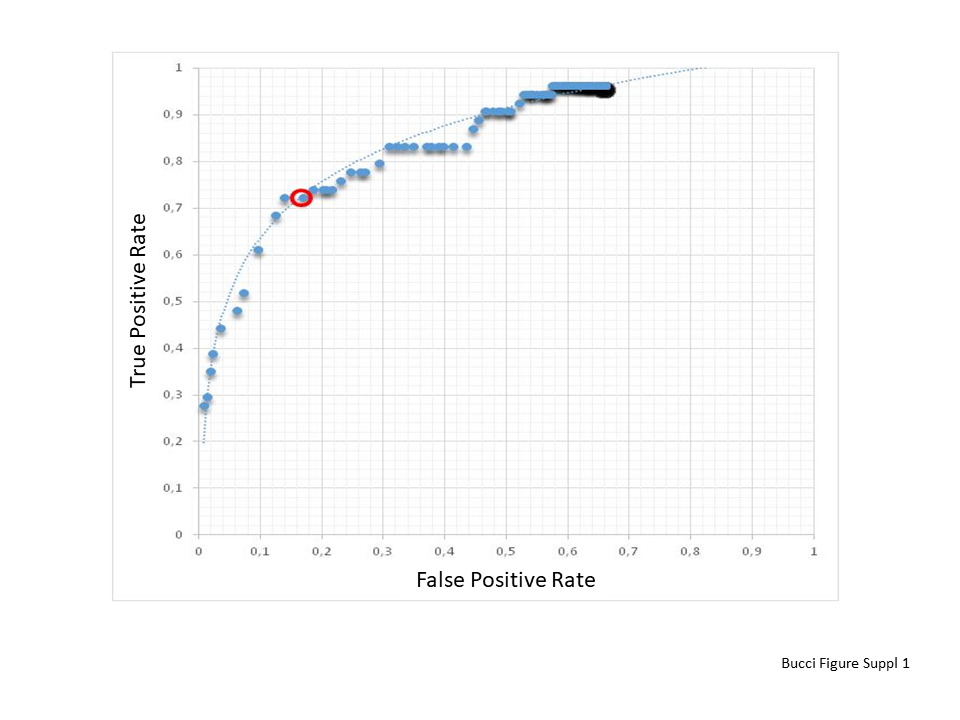

Supplement: Supplementary file 2 — Supplementary Figure 1(TIF 324 kb) [file 41419_2018_430_MOESM2_ESM.tif]
