# Supplementary material for: Automatic detection of image manipulations in the biomedical literature
Source: Cell Death Dis. 2018 Mar 14;9(3):400. doi: 10.1038/s41419-018-0430-3 (PMC5852055; doi:10.1038/s41419-018-0430-3)
Supplement: Supplementary file 3 — Supplementary material(DOCX 19 kb) [file 41419_2018_430_MOESM3_ESM.docx]

## Supplementary Materials

1. **R.O.C Analysis**

To check for the accuracy of the full pipeline, we let a human expert annotate all the 4778 image panels for the following features:

1. Being a software true positive (i.e. an image containing either a duplicated panel or some cloned features)
2. Being a software true negative (i.e. an image with no obvious manipulations included in the previous two categories)
3. Being a false positive (i.e. an image which was flagged by the software for containing either a duplicated panel or a cloned feature, which however turned to be clean)
4. Being a false negative (i.e. an image containing an undetected duplicated panel or cloned feature)

For the considered paper set, by varying the software parameters a direct “human vs machine” comparison was obtained. The corresponding R.O.C. analysis is represented in figure S1.

In figure S1, the red circle represents the True Positive Ratio and the False Positive Ratio obtained setting the software so that if an image contains two portions with an average normalized pixel by pixel difference in intensity (gray levels) equal to or within 2%, that image is flagged as potentially manipulated. The plot implies that using the selected software configuration, 28% of the overall manipulated images are expected to pass the software test as undetected, while 18% of the overall clean images are erroneously flagged by the software as manipulated. Since the actual fraction of manipulated images is low, the overall advantage in using the software mainly consists in reducing by about 5-fold the number of images that need to be checked for potential manipulations. Moreover, since the software highlights the potential manipulation by outlining the area in the image, human checking is much easier, and the time spent in this activity decreases correspondingly.
